# Illuminating cellular formaldehyde

**DOI:** 10.1038/s41467-020-20758-0

**Published:** 2021-01-25

**Authors:** Carla Umansky, Agustín E. Morellato, Lucas B. Pontel

**Affiliations:** Instituto de Investigación en Biomedicina de Buenos Aires (IBioBA), CONICET - Partner Institute of the Max Planck Society, C1425FQD Buenos Aires, Argentina

**Keywords:** Sensors and probes, Mechanisms of disease

## Abstract

Writing in Nature communications, Zhu and collaborators reported the development of a genetically encoded sensor for the detection of formaldehyde in cells and tissues. This tool has great potential to transform formaldehyde research; illuminating a cellular metabolite that has remained elusive in live structures.

## Formaldehyde, a ubiquitous and reactive metabolite

**Fig. 1:**
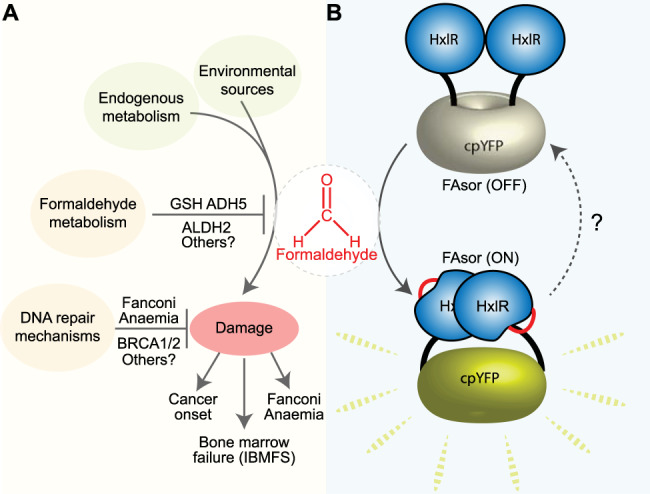
FAsor illuminates cellular formaldehyde. **A** Representation of cellular formaldehyde (FA) flux from environmental and endogenous sources (green circles). Light yellow circles represent the main mechanisms that counteract FA toxicity, including FA metabolism [ADH5, Glutathione (GSH) and ALDH2], and DNA repair mechanisms (Fanconi Anaemia DNA repair and the tumour suppressors BRCA1 and BRCA2). FA inflicted damage might underly the human condition Fanconi Anaemia, cancer onset and a type of inherited bone marrow failure syndrome (IBMFS), among others. **B** Scheme depicting FAsor, which consists of a single-chain protein formed by two HxlR modules separated by a cpYFP unit (HxlR-cpYFP-HxlR). In every HxlR module, FA crosslinks a lysine and a cysteine residue present in the first alpha helix (solid red lines), triggering a large conformational change that affects the spectral properties of the cpYFP unit. This change can be recorded ratiometrically as an increase in the fluorescence above 530 nm when excited at ~405 nm and a decreased when excited at ~488 nm. Dashed black line represents the still unresolved reversibility of FAsor.

Cells obtain energy and molecules required for sustaining life from multiple interconnected biochemical reactions known as metabolic pathways. These reactions can also generate additional reactive metabolites that might damage key biomolecules such as DNA and proteins. One toxic metabolite is formaldehyde (FA), the simplest and one of the most reactive aldehydes. FA is generated endogenously from vital pathways such as protein and nucleic acid demethylation, the one-carbon cycle and from the oxidative degradation of the one-carbon carrier tetrahydrofolate (THF) and some of its derivatives, among others^1^. Dietary supplements like the sweetener aspartame or fruit juices rich in methyl-pectins can be metabolized to methanol, which is further oxidized to FA in the liver^2^. Furthermore, pollution, cigarette smoke and certain chemicals are common environmental sources of FA, likely contributing to the ~50 micromolar FA concentrations reported in human blood (Fig. 1a)^2,3^.

FA might be implicated in cancer development in patients carrying mutations in the tumour suppressor BRCA2, and in the onset of some human conditions such as Fanconi Anaemia. This rare genetic disorder originates from mutations in genes coding for factors that repair DNA-interstrand crosslinks, one of the lesions inflicted by FA on the DNA (Fig. 1a)^4,5^. The World Health Organization (WHO) recognizes FA as an environmental human carcinogen, described to increase the risk of developing nasopharyngeal cancer^6^. Consequently, FA levels inside cells need to be strictly controlled by specific mechanisms that metabolize it into less reactive molecules, such as formate. Cytosolic FA metabolism initiates with the cellular antioxidant glutathione (GSH), which spontaneously reacts with FA producing S-hydroxymethyl-GSH (HSMGSH)^7^. This compound is oxidized by the enzyme Alcohol dehydrogenase 5 (ADH5), thus keeping a low – still unknown – intracellular concentration of free FA, and limiting redox balance disruption by recycling GSH^7,8^.

## Chasing in the gloom

A few methods including colorimetric assays, chromatography and mass spectrometry have been developed to measure FA in biological samples. However, these techniques often require sample destruction and time-consuming sample preparation. Hence, the in vivo study of FA has remained limited due to the lack of tools that are able to detect its real-time concentration and location in a non-invasive manner. Recently, some small fluorescent chemical molecules have been developed to measure FA both in vitro and in living cells. For example, the Aza-Cope-based probes are highly sensitive and specific to FA; however, their reaction kinetics are slow and irreversible^9^. Other probes, based on formamine and aminal groups^10^, react rapidly and reversibly with FA, but with the disadvantage that cells need to be pre-loaded with the probes for the experiments to be carried out. Moreover, directing these chemicals to subcellular organelles is still challenging, critically impairing their use in live imaging experiments.

Protein-based sensors have proven successful to monitor metabolites such as H_2_O_2_ or calcium in live cells and organisms^11,12^. The rationale behind these sensors is the ability of a metabolite to induce a conformational change in a specific protein that is fused to a circular permuted fluorescent protein (cpFP). This cpFP is not a classical fluorescent protein, but a version whose spectral properties are highly sensitive to conformational changes. Therefore, fusing a cpFP to a protein that undergoes a conformational change upon binding a metabolite might create a fluorescent sensor for the specific detection of that metabolite. This approach was used by Zhu and collaborators to develop a genetically encoded FA sensor, denominated FAsor (Fig. 1b)^13^.

## FAsor comes to shed light into formaldehyde biology

Zhu and collaborators started by characterizing a DNA transcriptional regulator from *Bacillus subtilis*, HxIR, that reacts with FA in a “gain of function” manner, driving gene expression in response to FA. Mechanistically, FA crosslinks the side chains of the cysteine and lysine residues present on the first alpha helix (Cys11 and Lys13 on helix α1). This reaction results in an intra-helical methylene bridge that, in turn, induces a large conformational change leading to the allosteric activation of HxIR. Authors hypothesized that this conformational change could be applied for developing a FA sensor by combining HxlR with a cpFP. Nine different chimeric proteins were generated fusing two HxlR monomers to a yellow cpFP (HxlR-cpYFP-HxlR or HYH). One of these variants showed a ratiometric FA response (HYH-5) – increased fluorescence emission at 530 nm when excited at ~500 nm and decreased fluorescence response when excited at ~405 nm, giving origin to FAsor (Fig. 1b).

FAsor can rapidly detect FA in vitro from concentrations as low as 20 µM and up to millimolar levels. Noteworthy, FAsor is not activated by acetaldehyde (AA), the second simplest aldehyde that differs from FA in having only one additional methyl group. In cells, FAsor is induced when the culture medium is supplemented with FA or THF, further supporting that THF degradation can be a source of FA^1^. Most importantly, FAsor can detect a rise in cellular FA upon the inhibition of ADH5, even in absence of FA in the culture medium, and in a physiological pH range. This finding sets out the grounds for future research using FAsor to study cellular FA in cells and living tissues, opening more opportunities for a better understanding of FA dynamics, subcellular distribution and FA interaction with other cellular components and compartments.

Remarkably, blocking ALDH2, a mitochondrial aldehyde dehydrogenase that can metabolize FA and AA, also induces the signal of FAsor, suggesting that ALDH2 operates to limit the concentration of endogenous FA. This result might have wide implications for human health considering more than 30% of East Asians and over 540 million people worldwide have a dominant negative mutation in *ALDH2* that reduces the activity of this enzyme (the ALDH2*2 polymorphism). The ALDH2*2 carriers develop facial flush symptoms after consumption of alcohol and present an increased risk of developing oesophageal cancer, which might be a consequence of higher intracellular FA levels. Interestingly, Asian patients carrying the ALDH2*2 flush mutation develop a severe bone marrow failure syndrome if they also harbour biallelic mutations in the gene coding for ADH5^14^. Indeed, the combined inactivation of *Aldh2* and *Adh5* in mice produces a phenotype that resembles this human condition^14^. Zhu et al. have shown that FAsor can be introduced into the dentate gyrus of wild-type mouse brains by viral transduction, and signal monitored in acute brain slices by two-photon microscopy. Thus, FAsor appears to be a promising tool for studying the biology underlying a condition characterized by the accumulation of cellular FA such as the above-mentioned deficiency in ALDH2 and ADH5.

One main drawback of FAsor in the context of FA biology is that it can also be activated by methylglyoxal (MG), which is a cellular aldehyde generated as a byproduct of glycolysis. This dual specificity was not surprising as HxlR responds to both FA and MG in *Bacillus subtilis*^15^, and indeed it sparks interesting biological questions, such as what is the relationship between FA and MG metabolisms, and whether it is possible to split FA from MG HxlR specificity, thus creating unique specific sensors for each of these aldehydes.

The use of FAsor for studying FA biology will require the careful design of controls to discern whether the signal originates from FA or MG. Nonetheless, with the adequate controls in place, FAsor could shed light into unknown cellular FA sources and be used to screen for enzymes that control cellular FA levels using widely available technologies such as confocal microscopy and flow cytometry. For example, in their paper, Zhu et al. showed that FAsor lights up upon blocking GSH metabolism, indicating that GSH limits intracellular FA concentrations. This result supports recent findings showing that GSH synthesis prevents FA cytotoxicity^7^. Furthermore, FAsor could be applied to screen for drugs or compounds able to modulate the cellular levels of FA with therapeutic purpose. These compounds would be particularly interesting for the treatment of cancers with mutations in some DNA repair factors such as BRCA1 or BRCA2, which have been shown to protect the genome from FA^1,5^.
